# Inside the Thrombus: Association of Hemostatic Parameters With Outcomes in Large Vessel Stroke Patients

**DOI:** 10.3389/fneur.2021.599498

**Published:** 2021-02-22

**Authors:** Juan Marta-Enguita, Manuel Navarro-Oviedo, Roberto Muñoz, Jorge Olier-Arenas, Guillermo Zalba, Ramon Lecumberri, Maite Mendioroz, Jose A. Paramo, Carmen Roncal, Josune Orbe

**Affiliations:** ^1^Laboratory of Atherothrombosis, CIMA-Universidad de Navarra, Instituto de Investigación Sanitaria de Navarra, IdisNA, Pamplona, Spain; ^2^Neurology Service, Complejo Hospitalario de Navarra, IdisNA, Pamplona, Spain; ^3^Red de Investigación Cooperativa de Enfermedades Vasculares Cerebrales (INVICTUS PLUS), Madrid, Spain; ^4^Radiology Service, Complejo Hospitalario de Navarra, IdisNA, Pamplona, Spain; ^5^Department of Biochemistry and Genetics, University of Navarra, IdiSNA, Pamplona, Spain; ^6^Haematology Service, Clínica Universidad de Navarra, Pamplona, Spain; ^7^Neuroepigenetics Laboratory-Navarrabiomed, Complejo-Hospitalario de Navarra, Universidad Pública de Navarra-UPNA, IdiSNA, Pamplona, Spain; ^8^CIBER Cardiovascular (CIBERCV), Instituto de Salud Carlos III, Madrid, Spain

**Keywords:** ischemic stroke (IS), thrombus, thrombin activatable fibrinolysis inhibitor (TAFI), matrix-metalloproteinase 10 (MMP-10), hemostasis

## Abstract

**Background:** Actual clinical management of ischemic stroke (IS) is based on restoring cerebral blood flow using tissue plasminogen activator (tPA) and/or endovascular treatment (EVT). Mechanical thrombectomy has permitted the analysis of thrombus structural and cellular classic components. Nevertheless, histological assessment of hemostatic parameters such as thrombin-activatable fibrinolysis inhibitor (TAFI) and matrix metalloproteinase 10 (MMP-10) remains unknown, although their presence could determine thrombus stability and its response to thrombolytic treatment, improving patient's outcome.

**Methods:** We collected thrombi (*n* = 45) from large vessel occlusion (LVO) stroke patients (*n* = 53) and performed a histological analysis of different hemostatic parameters [TAFI, MMP-10, von Willebrand factor (VWF), and fibrin] and cellular components (erythrocytes, leukocytes, macrophages, lymphocytes, and platelets). Additionally, we evaluated the association of these parameters with plasma levels of MMP-10, TAFI and VWF activity and recorded clinical variables.

**Results:** In this study, we report for the first time the presence of MMP-10 and TAFI in all thrombi collected from LVO patients. Both proteins were localized in regions of inflammatory cells, surrounded by erythrocyte and platelet-rich areas, and their content was significantly associated (*r* = 0.41, *p* < 0.01). Thrombus TAFI was lower in patients who died during the first 3 months after stroke onset [odds ratio (OR) (95%CI); 0.59 (0.36–0.98), *p* = 0.043]. Likewise, we observed that thrombus MMP-10 was inversely correlated with the amount of VWF (*r* = −0.30, *p* < 0.05). Besides, VWF was associated with the presence of leukocytes (*r* = 0.37, *p* < 0.05), platelets (*r* = 0.32, *p* < 0.05), and 3 months mortality [OR (95%CI); 4.5 (1.2–17.1), *p* = 0.029]. Finally, plasma levels of TAFI correlated with circulating and thrombus platelets, while plasma MMP-10 was associated with cardiovascular risk factors and functional dependence at 3 months.

**Conclusions:** The present study suggests that the composition and distribution of thrombus hemostatic components might have clinical impact by influencing the response to pharmacological and mechanical therapies as well as guiding the development of new therapeutic strategies.

## Introduction

Stroke is the primary neurovascular disease, being the second cause of death and disability worldwide (5.5 million deaths each year and 176.4 million stroke-related disabled people) with almost 14 million new cases around the world every year (1). Stroke severely hampers the normal daily activities of survivors affecting health and social-care resources (2). Moreover, in 2047, the number of stroke events is expected to increase in almost 40,000 incident strokes and 2.58 million prevalent cases in Europe, in part as a consequence of the aging of the population (3).

Ischemic stroke (IS) accounts for the majority of strokes and in caused by the presence of a thrombus or an embolus in brain vessels. The current goal for the management of IS is based on the restoration of the cerebral blood flow achieved by the use of the thrombolytic drug, tissue plasminogen activator (tPA), and/or endovascular treatment (EVT) to remove thrombi (4). The successful introduction of endovascular thrombectomy procedures within the last decade has allowed thrombus retrieval and its detailed analysis. The study of thrombi is crucial to understand diagnosis, treatment, and secondary prevention of acute IS and to design safe and efficient thrombolytic strategies to improve recanalization and prognosis of IS patients.

Several studies of IS thrombi have focused on their structural and cellular components (5). Among them, platelets and von Willebrand factor (VWF) are important factors in thrombus formation and have previously been shown as key components of acute IS thrombo-emboli (6). Erythrocite dominancy in thrombi has been associated with arterial thrombi from noncardiac source, whereas fibrin/platelet dominancy has been described as related to cardiac thrombi (7–9). Leukocytes are often present in thrombus and seem to be more dominant in cardiac thrombi (7, 8). However, when T cells were analyzed separately by CD3+ immunostaining, the number of T cells was significantly higher in atherothrombotic thrombi than in thrombi from patients with cardioembolic or other causes stroke (10).

In search of new pharmacological alternatives for patients that do not benefit from current therapies, preclinical studies are exploring the potential of new thrombolytic compounds in different models of IS. Specific inhibitors of antifibrinolytic proteins are under development as the diabody against plasminogen activator inhibitor-1 (PAI-1) and thrombin-activatable fibrinolysis inhibitor (TAFI) (11). This simultaneous inhibition of TAFI and PAI-1 showed increased profibrinolytic effects without adverse bleeding (12). Moreover, already approved drugs, as the mucolytic drug N-acetylcisteine, by dissolving the disulfide bonds of large VWF multimers, have been proven to accelerate thrombus dissolution and prevent rethrombosis in rodent models of IS resistant to tPA (13). In line with these results, a disintegrin and metalloproteinase with a thrombospondin type 1 motif member 13 (ADAMTS13), which cleaves VWF, dissolves the t-PA-resistant thrombi. Consequently it reduces cerebral infarct sizes showing a potent thrombolytic activity in experimental models of stroke (14). Finally, matrix metalloproteinases (MMPs) could also play a role in thrombolysis, since the fibrinolytic and the MMPs systems cooperate in thrombus dissolution by acting on fibrin(ogen) directly or by collaborating with plasmin. Precisely, plasmin is able to cleave and activate several MMPs (MMP-1, MMP-3, and MMP-9) that can take part in the dissolution of the fibrin clot directly or interacting with other elements of the fibrinolytic system (15, 16). Specifically, our group has shown the fibrinolytic role of MMP-10 by preventing the activation of TAFI (17). We have reported that the administration of MMP-10 is as efficient as tPA reducing infarct size and demonstrated that a combination of MMP-10 with tPA achieves further reduction in brain damage by blocking tPA-induced neuronal excitotoxicity in IS experimental models (18).

The histological location of TAFI and MMP-10 in stroke thrombi still remains unknown, and their presence could determine thrombus stability and the response to thrombolytic therapy. In this study, we therefore collected thrombi retrieved from large vessel occlusion (LVO) stroke patients and subjected them to histological assessment of different hemostatic parameters with a specific focus on TAFI and MMP-10. Furthermore, we investigated their association with clinical outcomes.

## Materials and Methods

### Study Population

A total of 53 serial acute LVO IS patients admitted to the Complejo Hospitalario de Navarra Stroke Unit who underwent EVT between November 2015 and November 2017 were recruited. Adequate and correctly processed histological material was available only from 45 patients. Depending on the degree of fragmentation, it was either collected in one piece or in multiple pieces. All collected material from the same patient was processed together as one. The decision to perform EVT, associated or not with intravenous tPA, was made according to guidelines at the time of patient admission as the standard of care for acute IS (19). Endovascular procedure was performed using a stent-retriever [pRESET (Phenox, Germany); Catch (Balt, France); Tigertriever and Comaneci (Rapid Medical, Israel)] or an aspiration device (Penumbra, Penumbra, USA) according to interventionalist's criteria.

### Clinical Information

Demographics (age, sex) and other baseline characteristics of the patients, including previous cardiovascular disease, vascular risk factors, systolic, and diastolic blood pressure (SBP and DBP, respectively) at admission, serum glucose, stroke severity assessed by the National Institutes of Health Stroke Scale (NIHSS), previous use of antithrombotic agents (antiplatelet agents and anticoagulants), and treatment with tPA, were recorded. Main vascular risk factors documented were the following: type-2 diabetes mellitus (use of antidiabetic drugs, a casual plasma glucose >200 mg/dl, or fasting blood sugar ≥126 mg/dl or HbA1c ≥6.5%), hypertension (patients taking antihypertensive drugs or with blood pressure >140/90 mmHg on repeated measurements), hypercholesterolemia [patients receiving lipid-lowering agents or with triglycerides ≥200 mg/dl, an overnight fasting cholesterol level ≥240 mg/dl, or low-density lipoprotein (LDL) cholesterol ≥160 mg/dl], and current cigarette smoking. Based on the Trial of Org 10172 in Acute Stroke Treatment (TOAST) classification (20), etiological subtypes of ischemic stroke were assesed. C-reactive protein (CRP) and plasma creatinine were measured with autoanalyzers (Architect i2000SR, USA, and Cobas C311, Roche, Germany, respectively).

Alberta Stroke Program Early CT Score (ASPECTS) was collected by two independent radiologists regarding a CT scan obtained at admission for all patients. A second CT scan was done at 24–48 h in all patients to identify hemorragic transformation and evaluate infarct area. A 1.5 MRI scan was obtained within 1 week of stroke onset if not contraindicated (when MRI was contraindicated, delayed CT scan was elective) to confirm IS. The recanalization after thrombectomy was evaluated by angiography during endovascular procedure using the modified treatment in cerebral infarction (mTICI) score.

### Histological Analysis

**Figure 1:**
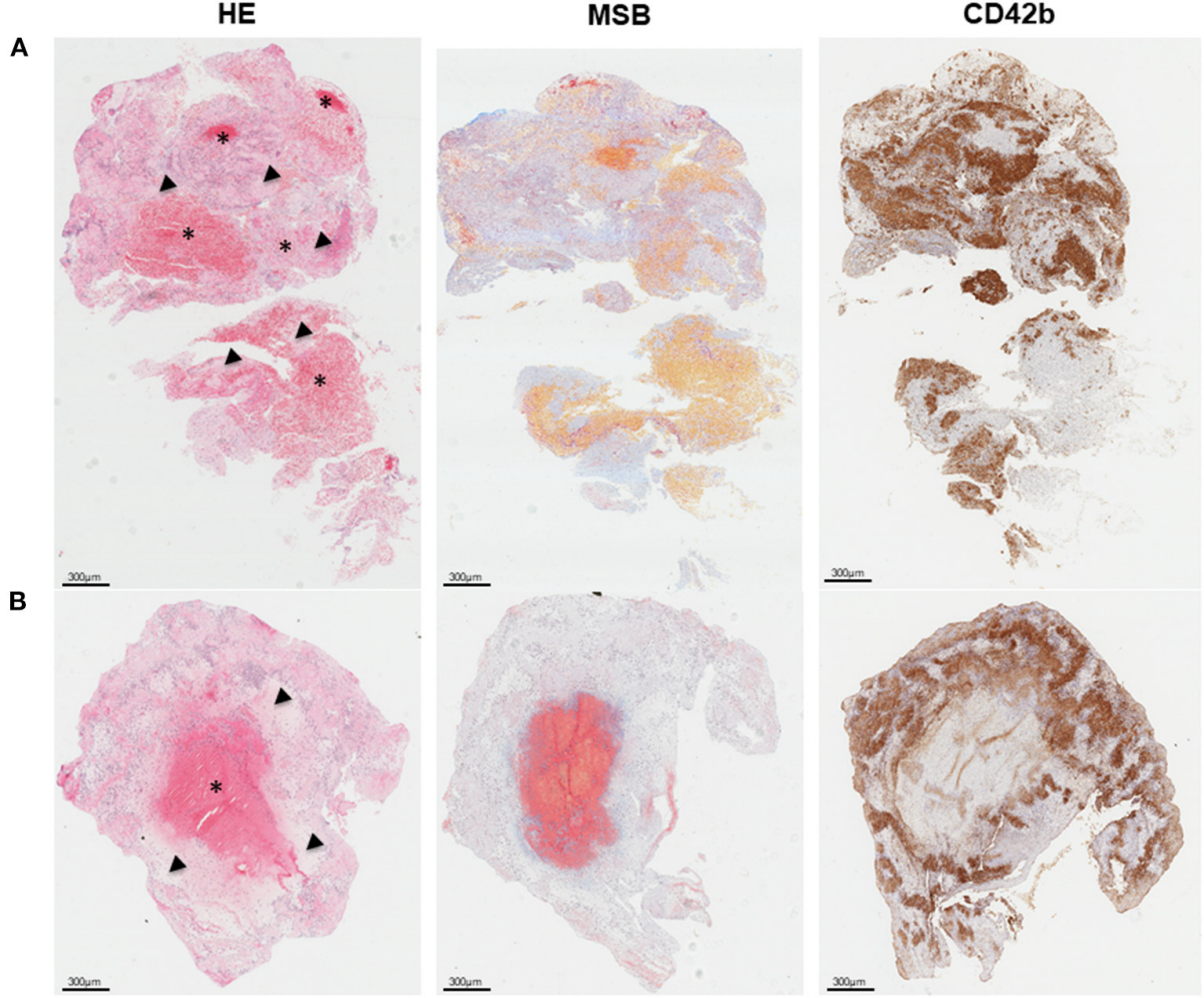
Overall thrombus composition and organization. **(A)** Red blood cell (RCB)-rich (asterisk) and platelet-rich areas (arrow head) are patchly distributed within thrombi. **(B)** Some thrombi present an RBC-rich core surrounded by platelet-rich material. From left to right, representative histological images of consecutive thrombi sections stained with hematoxylin and eosin (H&E), allowing identification of fibrin/platelet aggregates (pink), RBC (red), and nucleated cells (dark blue); Martius Scarlet Blue (MSB) showing the presence of fibrin (dark pink/red), RBC (yellow), and collagen (blue); and CD42b (brown) for platelets. Scale = 300 μm.

Retrieved thrombi (*n* = 45) were immersed in a saline solution and fixed for 24 h in formalin (PanReac AppliChem, Spain). Later, samples were embedded in paraffin by a tissue automatic processor (Tissue-Tek VIP, Sakura, Japan), and 3-μm sections of clot material were cut with a rotatory microtome (HM-340E, Microm, Germany). Serial slides from each thrombus were stained with Martius Scarlet Blue staining (Atom, UK), hematoxylin and eosin (PanReac AppliChem, Spain), and platelets glycoprotein Ib (CD42b, 42C01, Invitrogen, USA) to visualize their general internal organization (Figure 1). H&E allows identification of platelet/fibrin aggregates (pink), red blood cells (RBCs, red), and nucleated cells (dark blue), whereas the presence of fibrin (dark pink/red), red blood cells (RBCs, yellow), and collagen (blue) was demonstrated selectively by Martius Scarlet Blue (MSB) staining. Moreover, specific antibodies against VWF (A0082, Dako), T lymphocytes (anti-CD3, A0452, Dako), leukocytes (antiCD45, NCL-LCA-RP, Leica Biosystems), macrophages (anti-CD68, M0814, Dako), TAFI (AP17235PU, Origene), and MMP-10 (OAAF01865, Aviva) were also assayed.

Deparaffined and hydrated slides were incubated with citrated antigen retrieval solution (pH 6.10, Dako) at 95°C for 20 min or with Tris–ethylenediaminetetraacetic acid (EDTA) pH 9 for CD3 immunostaining (Master Diagnostica). Then, endogenous peroxidases were blocked with 5% hydrogen peroxide for 20 min at room temperature (RT) in the dark. Slides were then washed in Tris saline buffer (TBS, pH 7.36, 25 mM Tris). Sections were blocked using normal goat serum (Dako) for 1 h at RT. Sections were then incubated overnight at 4°C, with the primary antibodies. After washing, slides were incubated with the required secondary antibodies using the anti-rabbit or anti-mouse Dako Envision System-HRP (Dako) for 30 min and developed with diaminobenzidine (DAB, Dako) followed by counterstaining with Harris' hematoxylin. Slides were then mounted with distyrene plasticizer and xylene mixture (DPX, VWR Chemicals).

Double immunofluorescence was performed to localize TAFI and MMP-10 with specific cell types in thrombi tissue. Briefly, slides were incubated with a mix of primary antibodies overnight at 4°C. After washing, slides were incubated with the corresponding secondary antibodies for 30 min, using a goat anti-rabbit Alexa fluor 488 antibody (Invitrogen) or a biotinylated goat anti-mouse antibody (Dako) that was amplified with the Cy3 NEL 704 kit (PerkinElmer). Finally, slides were mounted with VECTASHIELD® Antifade Mounting Medium on DAPI (Novus Biological). Double immunofluorescence for TAFI and MMP-10 was performed with the rabbit anti-TAFI antibody described above and a monoclonal anti-MMP-10 (MAB9101, R&D systems).

Immunostained slides were subsequently scanned (Aperio ImageScope, Leica ByoSistems, Germany and Vectra Polaris, Perkin Elmer, USA) and quantified with ImageJ software (21). The percentage of positively stained area in total tissue area is presented as representative of thrombi content for TAFI, MMP-10, VWF, fibrin, RBC, and platelets (CD42b), whereas the positive cell number per square millimeter is given for nucleated cells (leukocytes, lymphocytes, and macrophages).

### Outcome Measures

Individual scores in the modified Rankin Scale (mRS) at 90 days, established by face-to-face interview with a stroke specialized neurologist, were the main clinical outcome. Other clinical outcomes included were as follows: (a) 3-month all-cause mortality; (b) 3-month functional independence (FI), categorized as 90-day mRS <3; (c) successful recanalization, defined as mTICI 2b or 3; and (d) hemorrhagic transformation after ischemic stroke according to the European Cooperative Acute Stroke Study III (ECASS III) classification (22), including hemorrhagic infarcts (HI type 1 or 2), parenchymal hematomas (PH type 1 or 2) and remote hematomas or subarachnoid hemorrhages.

### Plasma Levels of VWF, MMP-10, and TAFI

Within the following 24 h after admission, venous blood samples were drawn from all patients and centrifuged at 1200 × *g* for 15 min within 2 h of collection and subsequently stored at −80°C for further analysis. VWF activity (Innovance VWFAc, Siemens, Spain), MMP-10 levels (R&D Systems, USA), and TAFI activity (TAFIa, STA STACHROM TAFI, Stago, France) were measured with an automated ELISA analyzer TRITURUS (Grifols, Spain) in citrated plasma samples after being thawed on ice and thoroughly vortexed. The detection limit of the assays was 2.2%, 15.1 pg/ml, and 5% for VWFAc, MMP-10, and TAFIa, respectively. All experiments were performed and analyzed in a blinded manner.

### Statistical Analysis

Normality of distributions was assessed graphically and with the Shapiro–Wilk test. Non-normally distributed variables were presented as median with interquartile range (IQR), while continuous variables with normal distributions were presented as mean with standard deviation (SD). Logarithmic transformation was applied for continuous variables with skewed distributions. An unpaired *t*-test or the Wilcoxon rank-sum test was applied to compare continuous variables between groups depending on their distribution. The chi-square test or, in the case of small-expected frequencies, Fisher's exact test were performed to compare binary categorical variables distribution between groups. Correlation between continuous variables was evaluated by pairwise Spearman correlation test. Association between MMP-10 and TAFI thrombi content was assessed by linear regression analysis. Based on TOAST criteria, stroke subtype classification was assessed, and dichotomized etiological groups were created. Three groups of stroke severity by NIHSS score were categorized [(0–7), (7–14), and (>14)], and analysis of variance and trend analysis were performed.

Selected multivariate binary logistic regression models were performed to evaluate associations between thrombi histological parameters and circulating measurements with clinical outcomes. Results were expressed as odds ratios (ORs) with 95% confidence intervals (95% CIs).

Statistical significance was considered for all analyses if *p* < 0.05. STATA software (version 16, StataCorp LLC, Texas, USA) was the statistic software for this study.

## Results

### Patients Clinical Characteristics

Fifty-three patients were finally included in the study. Clinical characteristics of the patients are shown in Table 1. Revascularization after EVT was achieved in 84.9% of patients treated. Stent-retriever devices were deployed in 71.2% of patients, and aspiration techniques alone were performed in the remaining 28.9% of patients. A high percentage of patients (71.7%) was treated also with intravenous tPA as IS standard of care when they did not have contraindication to tPA treatment. Median time from stroke onset to EVT treatment was 190 min (IQR, 150–240) with an onset-to-needle time for those with intravenous fibrinolysis of 86 min (IQR, 65–130). No differences in onset-to-femoral puncture time was observed in those patients who received tPA vs. those without tPA treatment [median, (IQR): 190 (155–265) vs 220 (140–232), *p* = 0.53]. Stroke severity was severe with a median NIHSS score of 18 points (IQR, 15–21). ICH incidence was 35.9% (18.9% PH), and mortality rate was 25%.

**Table 1:** Patients clinical characteristics.

**Variable**	***n* = 53**
Age, years^†^	74.6 (61.9–78.2)
Female, *n* (%)^†^	25 (47.2)
Hypertension, *n* (%)^†^	31 (58.5)
Type-2 Diabetes, *n* (%)^†^	10 (18.9)
Dyslipidemia, *n* (%)^†^	31 (58.5)
Antiplatelet therapy, *n* (%)^†^	12 (22.6)
Anticoagulant therapy, *n* (%)^†^	18 (34.0)
SBP at admission, mmHg^*^	143.5 (23.7)
DBP at admission, mmHg^*^	82.0 (15.1)
Serum glucose at admission, mg/dL^†^	115 (98–140)
Neutrophil count at admission, × 10^9^/L^†^	5.7 (4.3–7.7)
Lymphocyte count at admission, × 10^9^/L^†^	1.7 (1.3–2.6)
Baseline NIHSS score^†^	18 (15–21)
ASPECTS, points^†^	10 (8–10)
Baseline mRS score, *n* (%)^†^	
mRS 0	34 (64.2)
mRS 1	10 (18.9)
mRS 2	9 (17.0)
Etiologic subtype by TOAST, *n* (%)	
Atherothrombotic^†^	8 (15.1)
Cardioembolic^†^	33 (62.3)
Undetermined^†^	10 (18.9)
Others^†^	2 (3.8)
Intravenous thrombolysis, *n* (%)^†^	38 (71.7)
Recanalization (TICI 2B-3), *n* (%)^†^	45 (84.9)
Hemorrhagic transformation, *n* (%)^†^	19 (35.9)
3-month mortality, *n* (%)^†^	13 (25)
3-month functional independence, *n* (%)^†^	24 (46.2)

*SBP, systolic blood pressure; DBP, diastolic blood pressure; NIHSS, National Institute of Health Stroke Scale; ASPECTS, Alberta Stroke Program Early CT Score; mRS, modified Rankin Scale; TOAST, Trial of Org 10172 in Acute Stroke Treatment; TICI, Treatment in Cerebral Infarction*.

* *Continuous variables with normal distributions are presented as mean (SD)*.

† *Continuous non-normally distributed variables are presented as median (IQR 25–75)*.

‡ *Categorical variables are presented as n (%)*.

### Histological Characteristics of Thrombi

Only 45 IS thrombi properly retrieved after thrombectomy were analyzed. According to usual description of thrombi microscopic distribution (23, 24), two different patterns are interspersed within the analyzed thrombi (Figure 1A): on the one hand, the RBC-rich areas, composed of packed RBC within a meshwork of fibrin and little or no nucleated cells; on the other hand, the platelet-rich areas with fibrin staining through the platelets region. From the 45 analyzed thrombi, this pattern could be identified in 23 with a wide heterogeneity in quantity and distribution of those regions. Some thrombi, however, mainly consisted of a RBC-rich core that was surrounded by a platelet-rich matrix (18/45) (Figure 1B).

**Figure 2:**
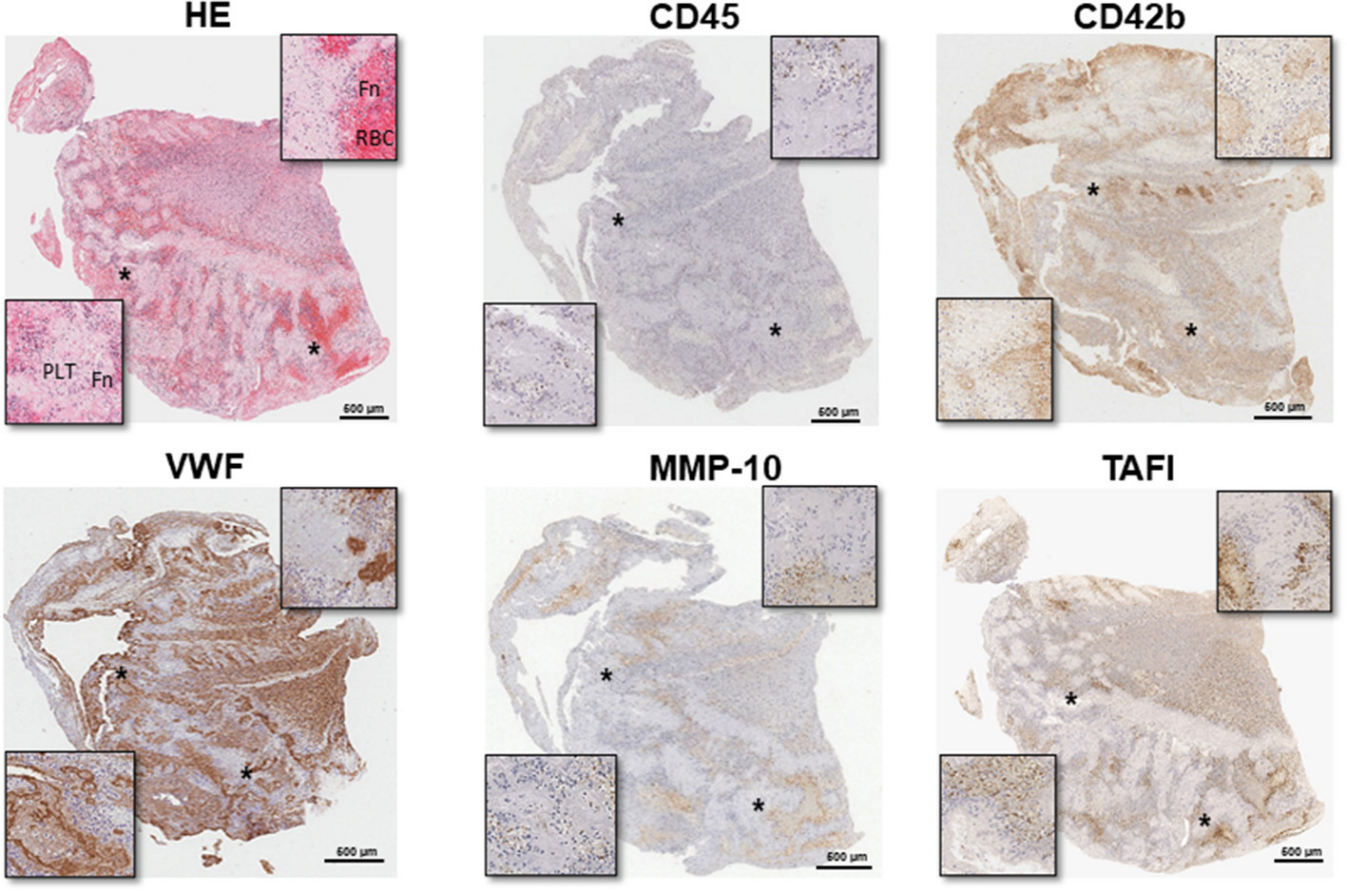
Location and distribution of different cellular and hemostatic parameters in retrieved stroke thrombi. Representative immunohistological images of consecutive thrombi sections stained with hematoxylin & eosin (H&E), allowing the identification of fibrin/platelet aggregates (pink), red blood cell (RCB) (red), and nucleated cells (dark blue); leukocytes (CD45), von Willebrand Factor (VWF), platelets (CD42b), matrix metalloproteinase-10 (MMP-10), and thrombin-activatable fibrinolysis inhibitor (TAFI), stained in brown. Magnification images of selected areas (*). Scale = 300 μm. Fn, fibrin; PLT, platelets; RBC, red blood cells.

As shown in Figure 2, leukocytes were mainly found at the interface between RBC- and platelet-rich areas but also within platelet-rich zones. Moreover, VWF staining was localized scattered in platelet-rich areas and through fibrin-positive regions.

To assess the relative contribution of each thrombus element, we quantified the stained area of RBC and fibrin (MSB), platelets (CD42b), and VWF. In addition, number of leukocytes (CD45), macrophages (CD68), and T lymphocytes (CD3) were assessed for all thrombi (Table 2). Overall, the median amount of RBC-rich material was 12.8% (IQR, 9.3–29.1), similar to fibrin [16.2% (5.4–41)], platelets [15.0% (4.6–26.7)], and VWF content [11.0% (6.3–17.4)]. A median of 308.9 leukocytes/mm^2^ (IQR, 216.3–513.3), 32.9 lymphocytes/mm^2^ (19.5–63.9), and 98.7 macrophages/mm^2^ (44.9–264.2) were observed within the thrombus.

**Table 2:** Quantification of hemostatic parameters in retrieved stroke thrombi.

**Thrombi content**	**% of stained Area**
RBC	12.8 (9.3–29.1)
Fibrin	16.2 (5.4–41)
Platelets (CD42b+)	15.0 (4.6–26.7)
VWF	11.0 (6.3–17.4)
TAFI	2.1 (0.9–3.8)
MMP-10	2.9 (0.15–8.1)
**Thrombi content**	**No. of cells/mm**^**2**^
Leukocytes (CD45+)	308.9 (216.3–513.3)
Macrophages (CD68+)	98.7 (44.9–264.1)
T Lymphocytes (CD3+)	32.9 (19.5–63.9)

*Data are presented as median (IQR 25–75)*.

*RCB, red blood cells; VWF, von Willebrand Factor; TAFI, thrombin-activatable fibrinolysis inhibitor; MMP-10, matrix metalloproteinase-10*.

As shown in Table 3, analysis of cell types and proteins content in stroke thrombi showed a positive correlation between fibrin and RBC (*r* = 0.38, *p* < 0.05), as well as with platelets (*r* = 0.36, *p* < 0.05). Meanwhile, VWF correlated with platelets (*r* = 0.32, *p* < 0.05) and leukocytes (*r* = 0.37, *p* < 0.05). After linear regression multivariate analysis of thrombi components, only the association between fibrin and platelets [B = −0.07 (−0.11–−0.03), *p* = 0.002], VWF and leukocytes [B = 6.46 (2.33–10.58), *p* = 0.003], and VWF and platelets [B = 0.23 (0.01–0.45), p =0.042] remained significant after adjusting for age and sex.

**Table 3:** Correlations between cell types and proteins content in stroke thrombi.

	**Fibrin**	**VWF**	**TAFI**	**MMP-10**
RBC (MSB)	*r* = 0.38^*^	*r* = 0.15	*r* = −0.22	*r* = −0.14
Platelets (CD42b+)	*r* = −0.36^*^	*r* = 0.32^*^	*r* = −0.01	*r* = −0.012
Leukocytes (CD45+)	*r* = −0.09	*r* = 0.37^*^	*r* = −0.18	*r* = −0.14
Macrophages (CD68+)	*r* = −0.18	*r* = −0.05	*r* = −0.05	*r* = −0.03
T Lymphocytes (CD3+)	*r* = −0.25	*r* = −0.10	*r* = −0.02	*r* = −0.02
Fibrin (MSB)				
VWF	*r* = −0.22			
TAFI	*r* = −0.24	*r* = −0.16		
MMP-10	*r* = −0.13	*r* = −0.30^*^	*r* = 0.41^**^	

*RCB, red blood cells; platelets; VWF, von Willebrand Factor; TAFI, thrombin-activatable fibrinolysis inhibitor; MMP-10, matrix metalloproteinase-10; MSB, martius scarlet blue*.

* p < 0.05;

** *p < 0.01*.

**Figure 3:**
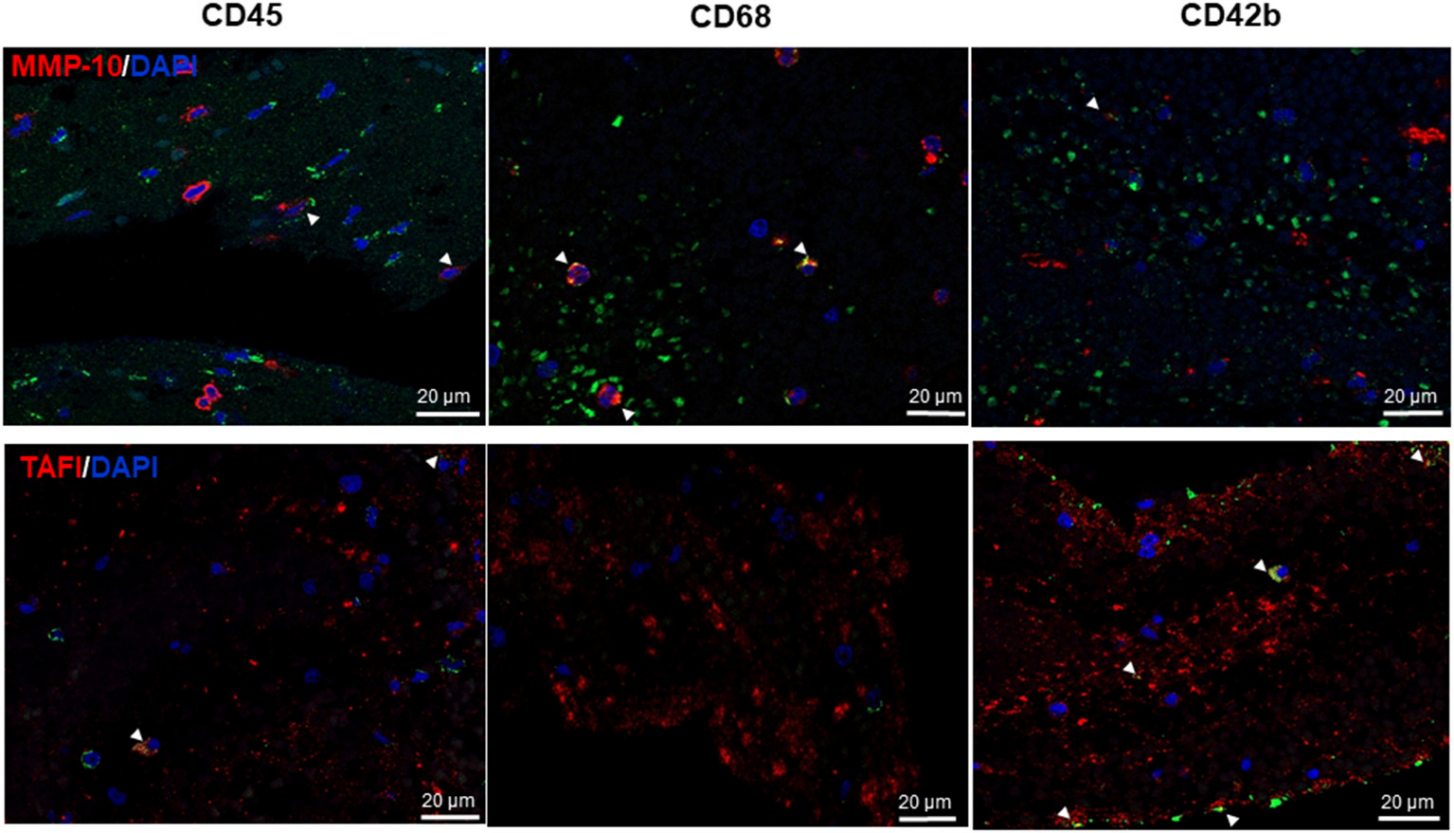
Matrix metalloproteinase-10 (MMP-10) and thrombin-activatable fibrinolysis inhibitor (TAFI) colocalize with inflammatory cells and platelets in thrombi. Double immunofluorescence for MMP-10 (top, red) and TAFI (bottom, red) and leukocytes (CD45, left), macrophages CD68 (middle), and platelets CD42b (right, green); cell nuclei are stained with 4′,6-diamidino-2-phenylindole (DAPI) (blue). Arrow heads point to double positive cells for MMP-10 (upper panels) and TAFI (lower panels) and the specified antigens (yellow). Scale = 20 μm.

**Figure 4:**
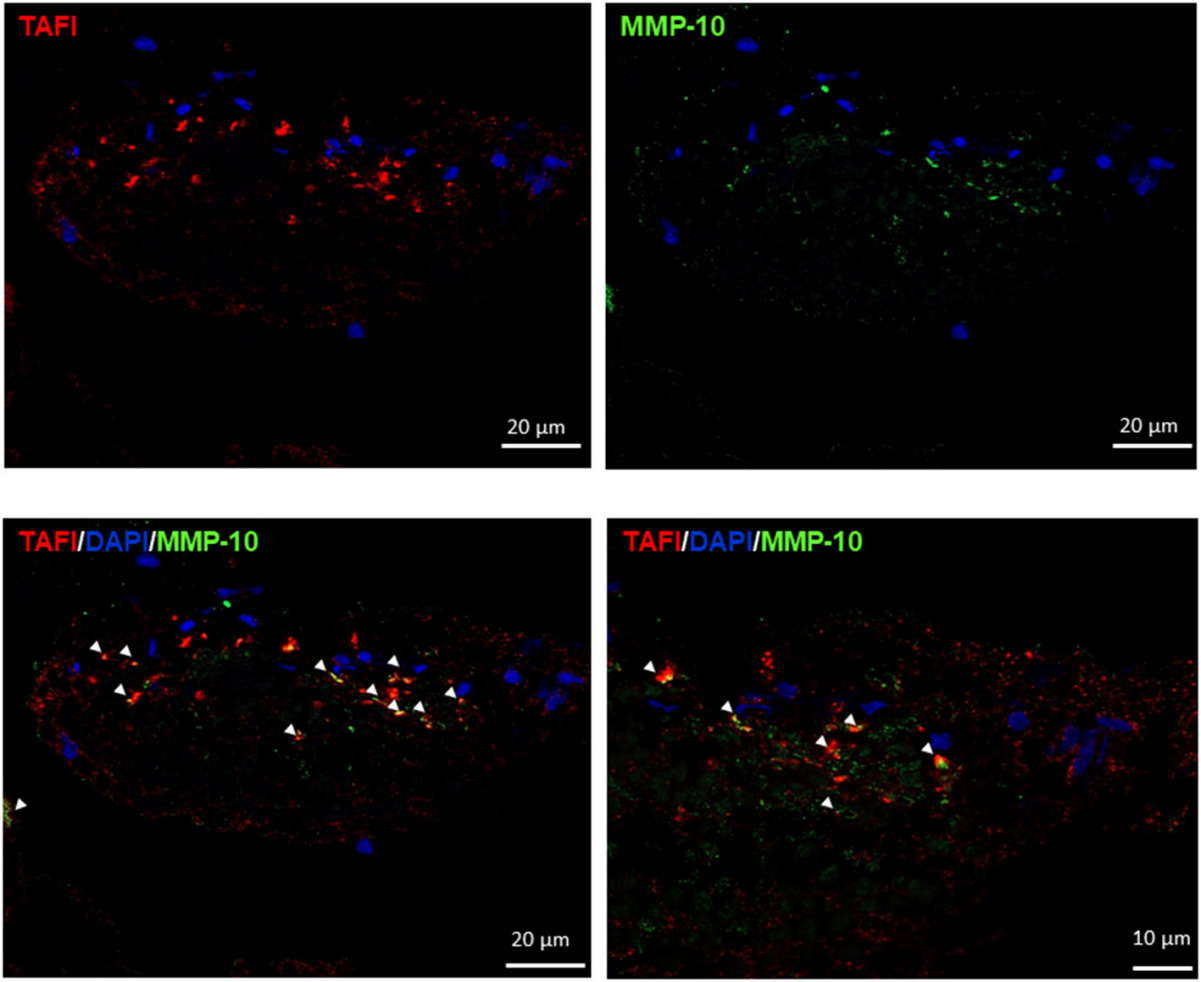
Matrix metalloproteinase-10 (MMP-10) and thrombin-activatable fibrinolysis inhibitor (TAFI) colocalization in thrombi. Immunofluorescence for TAFI (red), MMP-10 (green), and 4′,6-diamidino-2-phenylindole (DAPI) (blue). Arrow heads point to double positive cells for MMP-10 and TAFI. Scale = 20 and 10 μm.

Interestingly, MMP-10 and TAFI proteins were present in all thrombi [median (IQR): 2.9% (0.15-8.1) MMP-10 and 2.1% (0.9–3.8) TAFI] related to leukocyte distribution and primarily found at the interface between RBC and platelet-rich areas (Table 2 and Figure 2). As shown in Figure 3, MMP-10 colocalized with CD68 and with some CD45- and CD42b-positive cells, while TAFI signal was observed in some leukocytes and in platelets. Double immunostaining for TAFI and MMP-10 confirmed the colocalization of both proteins in thrombi (Figure 4).

Finally, MMP-10 staining positively correlated with TAFI (*r* = 0.41, *p* < 0.01) and negatively with VWF (*r* = −0.30, *p* < 0.05), while no association was found between thrombus TAFI and their cellular components or other analyzed proteins, except for MMP-10 (Table 3). This association between thrombus TAFI and MMP10 remained significant after adjusting for age and sex [B = 0.88 (0.55–1.20), *p* < 0.001].

### Association of Thrombi Components With Clinical Outcomes

**Figure 5:**
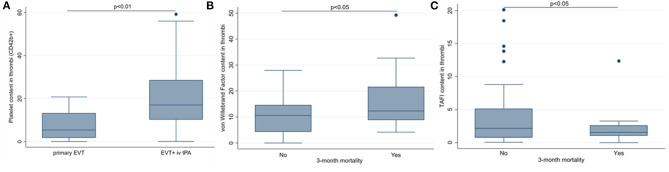
Association of thrombi components with clinical outcomes. **(A)** tissue plasminogen activator (tPA) treatment was associated with higher platelets (CD42b) content. **(B)** Patients who died within 3 months had higher von Willebrand Factor. **(C)** Patients who died within 3 months had lower thrombin-activatable fibrinolysis inhibitor (TAFI) content thrombi. EVT, endovascular treatment. Median (%) and interquartile range (IQR) 25–75, *p* < 0.05 and *p* < 0.01 using Chi-square or Fisher's exact test.

We further analyzed the association of thrombi components with clinical data. We found that the pharmacological intervention with tPA was associated with higher thrombi platelets content in univariate [median (IQR): 17.0% (10.1–29.4) vs 5.4% (1.5–14.8), *p* < 0.01, Figure 5A], and multivariate analysis [OR 1.23 (1.03–1.48), *p* < 0.05] after adjustment for confounding factors (baseline mRS).

None of the analyzed thrombus components were associated with complete recanalization after endovascular procedure, but the number of patients without recanalization was small (*n* = 8). Nevertheless, higher frequency of recanalization after the first pass of the device was associated to reduced macrophage content in thrombi [48.9 macrophages/mm^2^ (29.0–173.0) 1st pass vs. 189.2 macrophages/mm^2^ (74.3–305.6) more than first pass, *p* = 0.04] and remained associated after multivariate analysis by age and sex [OR (95% CI): 0.44 (0.20–0.97), *p* < 0.05]. Other studied components (platelets, leukocytes, T lymphocytes, fibrin, RBCs, VWF, TAFI, or MMP10) were not associated with recanalization or device passes.

No significant association between functional independence (FI) 3 months after stroke and content in thrombus for any of the studied thrombi components was observed (data not shown). Regarding 3-month mortality, patients who died within 3 months had higher VWF staining in thrombi [12.3% (8.9–21.7) vs. 10.6% (4.3–14.6), *p* < 0.05, Figure 5B]. Multivariate analysis adjusting for confounding factors (age and SBP) showed that thrombus VWF remained statistically significantly associated with mortality [OR (95% CI): 4.5 (1.2–17.1), *p* = 0.029]. In contrast, the amount of TAFI in thrombi was associated with lower mortality [1.5% (1.0–2.6) vs. 2.2% (0.7–5.1), Figure 5C] even after adjustment for age, glucose, and stroke severity [OR (95% CI): 0.59 (0.36–0.98), *p* = 0.043].

Moreover, a higher presence of leukocytes in thrombus was observed in those patients who died 3 months after stroke [444.7 (273.7–634.8) vs. 294.8 (191.8–439.0) leukocytes/mm^2^, *p* < 0.05], and it remained significant after adjustment by stroke severity and age [OR (95% CI): 4.98 (1.01-24.58), *p* < 0.05]. No association of 3-month mortality with other components of thrombus was observed.

Stroke etiological subtypes according to TOAST criteria and hemorrhagic transformation were also assessed in our cohort, and the associations with thrombi components were evaluated, but no association was found.

### Association of Circulating Hemostatic Parameters and Clinical Outcomes

When evaluating circulating levels of VWFAc, MMP-10, and TAFIa, no correlation with their thrombus content was found (Table 4) and only an association between VWFAc and thrombus lymphocytes was observed (*r* = 0.44, *p* < 0.01). Higher levels of circulating VWFAc were found in patients treated with tPA [60.3% (40.4–140.2) vs. 41.1% (25.3–50.2), *p* < 0.03] and in patients with worse clinical stroke severity by NIHSS score (ANOVA linear trend *p* < 0.001) with median values of NIHSS 0–7 [20% (15.6–35.3)], NIHSS 7–14 [48% (40.4–53.7)], and NIHSS >14 [57.5% (36.8–126.1)].

**Table 4:** Correlations between elements in stroke thrombi and circulating parameters.

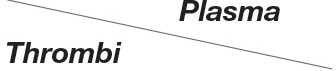	**VWF**	**TAFIa**	**MMP10**	**Platelets**	**Neutrophils**	**Lymphocytes**
RBC (MSB)	*r* = 0.17	*r* = 0.12	*r* = −0.10	*r* = −0.16	*r* = −0.09	*r* = 0.05
Platelets (CD42b+)	*r* = 0.10	*r* = −0.07	*r* = 0.13	*r* = 0.31	*r* = 0.09	*r* = 0.30
Leukocytes (CD45+)	*r* = −0.01	*r* = 0.04	*r* = 0.20	*r* = −0.21	*r* = −0.15	*r* = 0.13
Macrophages (CD68+)	*r* = 0.05	*r* = −0.05	*r* = −0.03	*r* = 0.20	*r* = 0.06	*r* = −0.04
T Lymphocytes (CD3+)	*r* = 0.44^**^	*r* = 0.15	*r* = 0.23	*r* = −0.06	*r* = −0.09	*r* = 0.05
Fibrin (MSB)	*r* = −0.11	*r* = −0.00	*r* = −0.05	*r* = −0.14	*r* = −0.04	*r* = −0.23
VWF	*r* = 0.19	*r* = −0.07	*r* = −0.12	*r* = 0.02	*r* = −0.15	*r* = 0.24
TAFI	*r* = 0.01	*r* = −0.03	*r* = −0.09	*r* = −0.08	*r* = −0.02	*r* = 0.28
MMP−10	*r* = −0.10	*r* = −0.21	*r* = −0.10	*r* = −0.12	*r* = 0.29	*r* = −0.23

*RCB, red blood cells; platelets; VWF, von Willebrand Factor; TAFI, thrombin-activatable fibrinolysis inhibitor; MMP-10, matrix metalloproteinase-10; MSB, martius scarlet blue*.

** *p < 0.01*.

As previously described, greater plasma levels of MMP-10 were associated with a decline in the glomerular filtration rate (*r* = −0.35, *p* < 0.05), increased C-reactive protein (CRP, *r* = 0.37, *p* < 0.05), and smoking habit [996 pg/ml [472–1150) vs. 359 pg/ml (264–620), *p* < 0.01]. Furthermore, we observed higher plasma levels of MMP-10 in patients with functional dependence [476 pg/ml (336–716) vs. 307 pg/ml (250–620), *p* = 0.026], which remained significant after adjustment for stroke severity and glucose [OR 5.13 (1.42–18.50); *p* = 0.013].

Finally, circulating TAFIa was associated with circulating platelets (*r* = 31, *p* < 0.05), and a trend between blood and thrombi platelets (*r* = 0.31, *p* = 0.061) was also observed (Table 4).

Neither of the studied circulating parameters (VWFAc, MMP10, and TAFIa) were associated with mortality after ischemic stroke in our cohort nor with stroke TOAST subtypes (data not shown).

## Discussion

In this study, we demonstrate the presence of MMP-10 and TAFI in all thrombi retrieved from LVO stroke patients at the interface between RBC and platelet-rich areas, matching leukocytes. Thrombus MMP-10 and TAFI content correlate independently of confounding factors, the local TAFI expression being significantly lower in patients who died within 3 months after stroke onset. Additionally, we show that thrombus MMP-10 inversely correlates with VWF content, which is also associated with 3-month mortality. Interestingly, the presence of platelets in the thrombus is associated with thrombolysis treatment as well as with thrombus VWF. Finally, plasma TAFI activity is associated with blood and thrombus platelets, whereas plasma MMP-10 is related to cardiovascular risk factors and 3-month functional dependence. Taken together, *in situ* analysis of different hemostatic and proteolytic parameters has prognostic implications in IS patients. These findings will help to understand thrombus stability and the response to IS therapies, leading to the development of individualized treatment strategies based on clot composition, which ultimately will improve patient outcome.

TAFI is a metallocarboxypeptidase activated by thrombin/thrombomodulin and plasmin that removes C-terminal lysine residues from partially degraded fibrin, preventing t-PA-plasminogen activation and inhibiting fibrinolysis. Previous reports showed the role of TAFI in the stabilization of newly formed fibrin clots (25). It was proposed that thrombin-induced activation of TAFI render newly formed fibrin clots more resistant to plasmin degradation (26). *In vivo* evidence for the role of TAFI in fibrinolysis was obtained in experimental venous and arterial thrombosis models using TAFI inhibitors (27–30). Decreased TAFI activity in rodent models of transient middle cerebral artery occlusion treated with TAFI inhibitor resulted in signs of lower microvascular thrombosis such as reduced fibrin deposition, regardless of infarct volume (29, 30). However, data from TAFI knockout mice indicated that TAFI deficiency did not have a significant impact on the rate of thrombus formation in arterial and venous thrombosis models (26, 31). Beyond fibrinolysis, TAFI also plays a role in inflammatory conditions, processing C-terminal arginine or lysine from bradykinin, complement factors C5a and C3a, etc., leading to a reduced inflammatory/immune response (32). In this regard, our group reported that TAFI deficiency increased brain damage and circulating microvesicles in IS model under thrombolysis, suggesting a higher inflammatory status in these mice (33). In line with these data, this study reports a significant association of thrombus TAFI with lower mortality, suggesting that TAFI could be implicated in IS at various levels, linking coagulation/fibrinolysis and the inflammatory/immune systems.

Furthermore, we previously demonstrated that MMP-10 cleaves TAFI, preventing its activation and enhancing tPA-induced fibrinolysis *in vitro* and in experimental models of thrombosis (17). In this study, we first identified TAFI and MMP-10 in human thrombi sections. Both proteins were localized in the same areas associated with leukocytes, and their stainings even colocalized in specific points of the thrombus surface, suggesting that the processing of TAFI by MMP-10 could be operational locally due to their proximity. Moreover, the strong correlation between both reinforces that their coexpression, at the surface of the thrombus, might favor TAFI inactivation by MMP-10 promoting thrombus lysis.

Interestingly, an inverse linear correlation was also observed between MMP-10 and VWF content, the latter previously associated with platelet-rich clots, dense fibrin structures, and poor revascularization outcome (6, 34). Our data suggest that higher expression of MMP-10 in thrombi might be associated with more effective fibrin lysis, lower VWF-fibrin structures, and better recanalization-related outcome. Moreover, we have also demonstrated an association between higher thrombus content of VWF and leukocytes with 3-month mortality in multivariate analysis. VWF is a large, multimeric glycoprotein that is crucial for normal hemostasis due to its role in the stable platelet plug formation at sites of vascular injury. Not surprisingly, different studies have identified VWF as an important constituent of stroke thrombi with a direct impact on thrombolysis (5, 6).

Next, we studied VWFAc, TAFIa, and MMP-10 in blood and their expression in thrombi. No significant correlation was found between circulating levels of studied proteins and their thrombus content, suggesting a different role of VWF, TAFI, and MMP-10 in circulation and locally, where they might be involved in thrombus formation and/or on cell-dependent thrombolysis. For instance, systemic VWFAc was associated with thrombus lymphocytes. The important role of immune cells on stroke progression is well established, likewise immune cells interact with molecules involved in platelet signaling, such as VWF, contributing to thrombus formation (35).

Furthermore, plasma TAFIa was correlated with platelets in the blood and thrombus. An association between higher plasma TAFI levels and the occurrence of IS was reported in a number of clinical studies (32–34, 36–38). It has been described that TAFI secreted upon platelet activation (39) might contribute to its variations in plasma. Our results support these data demonstrating a correlation between plasma TAFI activity and circulating platelets and locally showing their colocalization in thrombi. Even if TAFI and platelet content in thrombectomies did not correlate, their association within thrombi might suggest a role of locally secreted platelet-derived TAFI in the systemic crosstalk between coagulation and fibrinolysis, protecting thrombus against lysis.

In addition, higher circulating VWFAc were found in patients treated with tPA and in those with greater stroke severity, supporting previous studies showing that increased VWF levels were associated with elevated baseline stroke severity (by the NIHSS score) (40, 41). Moreover, elevated VWF antigen concentrations immediately after and 24 h postthrombolysis have also been associated to poor functional outcomes 3 months after ischemia (41), and tPA has been shown as potentially implicated with brain microvascular endothelial injury during postischemia in experimental models (42). Thus, it could be hypothesized that the increased levels of VWFAc after thrombolysis could be due to increased VWF antigen following endothelial damage caused by the thrombolytic agent.

Moreover, patients treated with tPA who underwent thrombectomy presented higher platelet fraction in thrombi. This fact has not been previously described but has been suggested in some studies (43). A paradoxical platelet activation has been reported secondary to fibrinolysis (44) as responsible for delayed thrombosis in some patients with tPA-resistant thrombi causing reocclusion and rethrombosis (45). Other additional mechanisms have been implicated in a higher platelets content in stroke thrombi of patients treated with tPA. An outer shell composed of platelets, extracellular DNA, and tight cross-linking of fibrin that confers resistance to fibrinolysis has been described in acute IS thrombi (46) and could support the higher platelet percentage found in thrombus of tPA-treated patients.

On the other hand, thrombus composition has been shown to be related to interventional times and efficacy of mechanical thrombectomy treatment for LVO stroke (9, 47). In this line, in our cohort, a higher macrophage presence in the thrombus was associated with lower frequencies of recanalization with the first pass of the device. There are previous data reporting that fibrin-organized thrombi need longer recanalization times (47) or a higher number of maneuvers during mechanical thrombectomy (9); thus, further studies are needed to analyze more deeply this association.

Finally, in this study, we observed an association of plasma MMP-10 levels with cardiovascular risk factors and 3-month functional dependence. In line with these results, we had previously reported that higher serum MMP-10 levels were associated with inflammatory markers and the presence of atherosclerotic plaques in asymptomatic subjects (48). Moreover, in IS patients, serum proMMP-10 concentration was independently associated with higher infarct volume, severe brain edema, neurological deterioration, and poor functional outcome at 3 months (49). Altogether, this study confirms that plasma MMP-10 might play a key role in cardiovascular diseases and therefore could be a potential biomarker for LVO stroke patients.

There are some limitations to this report that are worth considering. First, the modest sample size and the retrospective analysis of prospectively collected data are important methodological shortcomings. Second, only thrombi from those LVO patients in whom the thrombus could be partially or totally retrieved were available for study, whereas not recovered clots or those clots dissolved after tPA treatment could not be studied, and this impedes evaluation of tPA susceptibility and thrombectomy resistance. Third, the observational study design and the use of correlations to evaluate the association between variables do not allow to establish causal relationship and is only a rough approach to probably complex interrelationships between components in thrombi.

## Conclusion

Histological structure of thrombi is crucial to better understand their pathogenesis, properties, and clinical management in IS. The present findings suggest that the histological composition and distribution of different thrombi hemostatic components have prognostic implications, and it would most likely determine the clinical impact of pharmacological and mechanical strategies in order to guide personalized therapies for stroke patients.

## Data Availability Statement

The raw data supporting the conclusions of this article will be made available by the authors, without undue reservation.

## Ethics Statement

The studies involving human participants were reviewed and approved by the ethics committee of the Navarra Government (84/2018). The patients or their legally authorized representative provided their written informed consent to participate in this study.

## Author Contributions

JM-E participated in the experimental work, analysis of data, and edited and reviewed the manuscript. MN-O participated in the design of the project, experimental work and wrote, reviewed, and edited the manuscript. RM participated in the design of the project, samples collection, and reviewed the manuscript. GZ, RL, MM, JO-A, and JAP participated in the design of the project and reviewed the manuscript. CR and JO-A were in charge of the whole project design, supervised the work, and wrote, edited, and reviewed the manuscript. All authors contributed to the article and approved the submitted version.

## Conflict of Interest

The authors declare that the research was conducted in the absence of any commercial or financial relationships that could be construed as a potential conflict of interest.
